# Genetically Encodable Scaffolds for Optimizing Enzyme Function

**DOI:** 10.3390/molecules26051389

**Published:** 2021-03-04

**Authors:** Yong Quan Tan, Bo Xue, Wen Shan Yew

**Affiliations:** 1 Synthetic Biology for Clinical and Technological Innovation, National University of Singapore, 28 Medical Drive, Singapore 117456, Singapore; tan.yongquan@u.nus.edu (Y.Q.T.); xuebo@nus.edu.sg (B.X.); 2 Synthetic Biology Translational Research Programme, Yong Loo Lin School of Medicine, National University of Singapore, 14 Medical Drive, Singapore 117599, Singapore; 3 Department of Biochemistry, Yong Loo Lin School of Medicine, National University of Singapore, 8 Medical Drive, Singapore 117597, Singapore

**Keywords:** protein shells, synthetic enzymology, synthetic biology, protein scaffold, nucleic acid scaffold

## Abstract

Enzyme engineering is an indispensable tool in the field of synthetic biology, where enzymes are challenged to carry out novel or improved functions. Achieving these goals sometimes goes beyond modifying the primary sequence of the enzyme itself. The use of protein or nucleic acid scaffolds to enhance enzyme properties has been reported for applications such as microbial production of chemicals, biosensor development and bioremediation. Key advantages of using these assemblies include optimizing reaction conditions, improving metabolic flux and increasing enzyme stability. This review summarizes recent trends in utilizing genetically encodable scaffolds, developed in line with synthetic biology methodologies, to complement the purposeful deployment of enzymes. Current molecular tools for constructing these synthetic enzyme-scaffold systems are also highlighted.

## 1. Introduction

Synthetic biology is largely focused on controlling and repurposing cellular and biochemical phenomena, generally at the molecular level, for applications that are potentially beneficial to society [1]. The study of enzymes is of particular interest to synthetic biologists, as enzymes can and have been utilized for the production of plant-derived pharmaceutical ingredients in microbial hosts (e.g., biosynthesis of opiates, cannabinoids, and taxanes) [2,3,4], environmental remediation (e.g., degradation and upcycling of plastic waste) [5,6], curbing antimicrobial resistance (e.g., biosynthesis of novel antimicrobial polyketides and non-ribosomally synthesized peptides) [7,8], and diagnostic purposes (e.g., glucose oxidase for monitoring glucose levels) [9]. The exponential increase in genomic and protein sequences made freely available has aided the search for enzymes to carry out chemical transformations that might find use in the areas of applications mentioned, among others [10]. Common bottlenecks in the translation of this wealth of genomic enzymological information into biotechnological applications include enzyme instability, lower activity when expressed in heterologous hosts, interference from host metabolism, and suboptimal metabolic flux [11]. Two established strategies to overcome these limitations are (i) rational design and (ii) directed evolution of enzymes to identify sequence variants that confer desired properties. Such properties include stability against denaturation induced by elevated temperatures, organic solvents or non-physiological pH, higher catalytic efficiency, and utilizing or generating noncanonical metabolites. The considerable list of achievements in using sequence space exploration for improving enzyme function and properties has been documented in other reviews [11,12,13].

**Figure 1:**
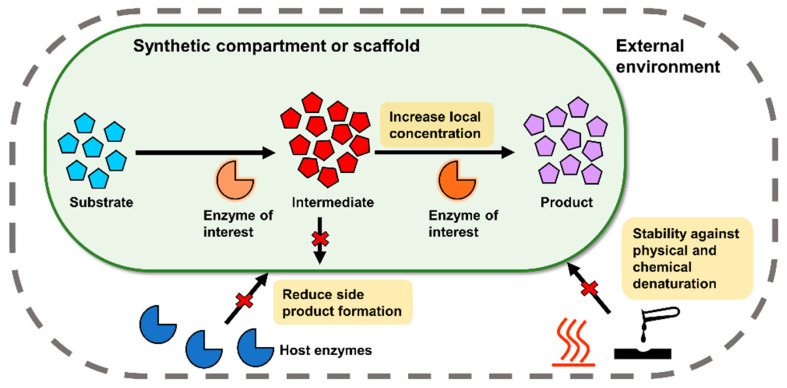
Enzyme compartmentalization or scaffolding has the potential to improve the efficiency of enzymes or metabolic pathways. Such strategies can also reduce the impact of interference from the external environment, either from the host cell or an ex vivo setting.

In recent years, there has been a growing awareness that regulating the microenvironment where enzymes and enzymatic pathways are deployed through the use of biomolecular scaffolds can also assist in relieving the aforementioned bottlenecks, providing a complementary approach to sequence space exploration [14]. Encapsulation of recombinant enzymes in thermostable protein shells has been demonstrated to confer resistance on the enzymes against chemical and physical insults [15,16,17,18]. Corralling of enzymes in a heterologous biosynthetic pathway into subcellular compartments has been reported to result in higher product(s) titers, conceivably due to improved substrate channeling (Figure 1) [19,20,21,22]. More recently, strategic installation of energy and signal emitting enzymes on scaffolds have improved their sensitivity and robustness for analytical applications [23]. While sequence space exploration has undoubtedly been successful in producing more robust and efficient enzymes, insights gained from one successful example are unlikely to be applicable to evolutionarily distant or unrelated enzymes due to the huge diversity of protein folds and domains [24]. Utilizing biomolecular scaffolds can provide a more generalizable route for imparting desired properties into enzymes. In addition, scaffolded enzymes can be recovered more easily in a bioprocessing setting, reducing cost in enzyme production [25]. The reductionist approach commonly taken in synthetic biology, where biological systems are reduced to their components based on individual function and recombined in a plug-and-play fashion, facilitates the juxtaposition between enzymes and unrelated scaffolds to synthesize new systems [26].

The information gleaned from decades of cellular and biochemical studies has shed some light on how and why cells spatially organize enzymes, in spite of the significant metabolic investment involved. Eukaryotic organelles have evolved to sequester specialized processes that might otherwise be incompatible with the bulk intracellular environment. Lipid-delineated organelles create a physicochemical boundary between the intra-organelle space and the external environment, preventing passive diffusion of macromolecules and most hydrophilic small molecules across the boundary. The endoplasmic reticulum provides a lipid-rich environment suitable for supporting membrane-associated enzymes that participate in the metabolism of lipids, lipoproteins, and hydrophobic xenobiotics [27]. The peroxisome, involved in the catabolism of fatty acids, encapsulates reactive oxygen species (ROS), which are generated during this process, in close proximity to superoxide dismutases that detoxify ROS [28]. This protects the rest of the cell from oxidative damage. Not all eukaryotic organelles are bordered by lipid membranes. In recent years, there has been an increasing recognition of protein condensates that phase separate from the aqueous cytoplasmic environment, exhibiting bulk physicochemical properties more akin to polar organic solvents than water [29]. These micron-scale proteinaceous condensates are termed membraneless organelles (MO) and are often found to harbor RNA and small nuclear ribonucleoproteins, increasing the rate at which RNA is processed.

The spatial optimization of enzymes is not the sole prerogative of the eukaryotic cell. Many bacterial and archaeal species produce supramolecular protein complexes, ranging from tens to hundreds of nanometers in scale, to perform specialized physiological functions, analogous to eukaryotic organelles. These include bacterial microcompartments (BMC) and encapsulins, found in both bacteria and archaea [30,31]. BMCs have been found to facilitate the carbon fixation step of photosynthesis and to catabolize short-chain alcohols, amines, and other niche carbon sources [30]. Some encapsulin shells have been observed to house enzymes involved in mitigating oxidative stress, suggesting these shells protect the host from harmful ROS that are released during these processes [31]. Understanding the characteristics and functions of naturally occurring compartments and scaffolds, present in all domains of life, sets the platform for hosting enzymes and improving their properties using either natural or engineered scaffolds.

The four main classes of biomolecules (proteins, nucleic acids, lipids, and carbohydrates) have been utilized, via synthetic biology and/or synthetic chemistry methodologies, as scaffolds for improving enzyme functionality. In this review, we focus on scaffolds that have been developed and enhanced using synthetic biology approaches. These are largely protein and nucleic acid-based due to their direct genetic manipulability and modularity. The in vivo use of lipid and polysaccharide scaffolds for hosting heterologous enzymes generally involves the co-opting of endogenous organelles and cellular structures, with limited modifications to these structures [20,32]. The in vitro use of lipid and polysaccharide/carbohydrate scaffolds is largely under the purview of synthetic chemistry [33,34]. Hence, lipid and carbohydrate-based scaffolds will not be discussed here.

This review summarizes recent advances in repurposing genetically encodable biomolecules as scaffolds for improving enzyme function for various applications in both intracellular and extracellular settings. Emphasis is placed on novel developments in the use of established scaffolds, and novel scaffolds with enzyme-related applications. Design principles pertinent to the creation and functionalization of these scaffolds are discussed. The studies highlighted demonstrate that modular biomolecular scaffolds are emerging platforms for optimizing enzyme function.

## 2. Protein Shells and Scaffolds

Proteins offer diverse molecular architectures to avail the creation of self-assembling structures that can encapsulate enzymes, as is the case for protein shells, or to spatially organize enzymes in a defined fashion, *vis-à-vis* protein scaffolds. A key advantage of protein compartments and scaffolds over lipid-delineated organelles is that protein assemblies can be expressed in heterologous hosts while retaining their biochemical properties. This provides a generalizable approach for harnessing their utility across various biotechnologically relevant prokaryotic and eukaryotic cell lines regardless of the origin of the protein compartment/scaffold. For example, the cyanobacterial carboxysome has been expressed in the tobacco plant to encapsulate the enzyme RuBisCO [35], while intrinsically disordered proteins (IDP) derived from arthropods are able to form MO in *Escherichia coli* [36]. The modular nature of protein shells and scaffolds offers tractability for bioengineering purposes.

**Figure 2:**
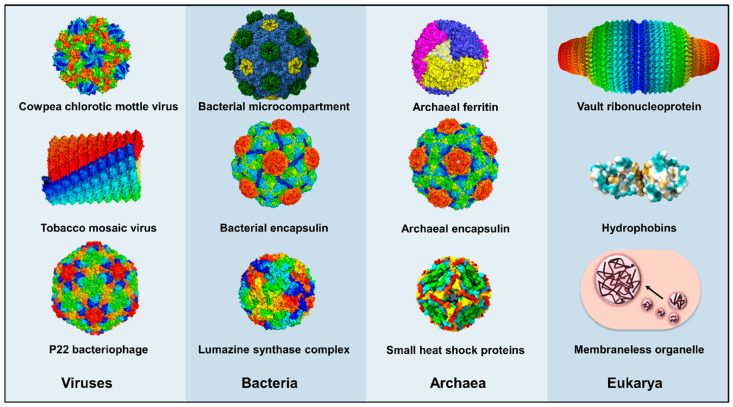
Structural models of some proteinaceous shells and scaffolds, from viruses and the three established domains of life, that have been engineered to host foreign enzymes. Surface representations are used for protein shells with known atomic-level structural models. For heteromeric shells, each component is colored differently to aid differentiation. For homomeric shells, selected polypeptide chains are colored discretely before applying appropriate symmetries to construct the intact shell. A hydrophobic surface representation is used for hydrophobins (cyan for hydrophilic, brown for hydrophobic). Atomic-level structures are not known for MOs, and a schematic of aggregation of spherical globules representing MO formation within a cell is shown. Models are not depicted to scale. The PDB accession codes used to generate these models are: 1ZA7 (cowpea chlorotic mottle virus [38]), 6R7M (tobacco mosaic virus [39]), 5UU5 (P22 bacteriophage [40]), 5V74 (bacterial microcompartment [41]), 4PT2 (bacterial encapsulin [42]), 1HQK (lumazine synthase [43]), 1S3Q (archaeal ferritin [44]), 2E0Z (archaeal encapsulin [45]), 1SHS (heat shock protein [46]), 4V60 (vault [47]), and 1R2M (hydrophobin [48]). Models were generated using UCSF Chimera and PyMOL [49,50].

**Figure 3:**
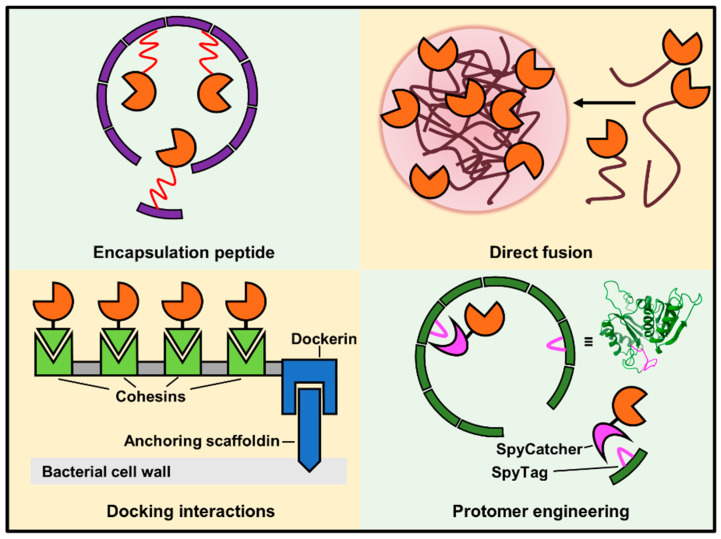
Examples of various methodologies for loading cargo (depicted by the orange circle with a missing slice) onto protein shells and scaffolds. Installation of a suitable encapsulation peptide sequence (red wavy line) on the cargo can direct the cargo into a shell (e.g., encapsulins, BMCs) [51,52]. Cargo can be translationally fused to a protein subunit of a compartment to direct encapsulation (e.g., MO) [36]. Use of docking/protein–protein interaction domains can direct cargo to the scaffold (e.g., cellulosome) [53]. The protomer of a shell can be engineered to present protein conjugation domains, such as SpyCatcher with SpyTag [54].

Protein shells and scaffolds are supramolecular structures, generally constructed from numerous polypeptide subunits (i.e., protomers), which span from approximately 10 to 1000 nm in size. Recent advances in structural determination of large protein complexes, especially in cryo-electron microscopy (cryo-EM) [37], have accelerated the elucidation of molecular interactions that contribute to the assembly of protein shells and scaffolds (Figure 2), facilitating protein engineering efforts. This section serves as a primer for recent developments in some of the myriad protein architectures that have been repurposed for supporting enzyme function, each with their distinct size (Table 1), composition, method of cargo loading (Figure 3), and biochemical properties.

### 2.1. Virus-Derived Protein Shells

Viruses possess a protein coat—the capsid—that wraps around the viral nucleic acid. Virus-derived structures that physically resemble viruses but lack the viral nucleic acid, and are, therefore, non-infectious, are termed virus-like particles (VLPs). Capsid structure can be broadly classified into those with regular geometries (e.g., icosahedrons and helices) or irregular geometries (e.g., complex or lipid-enveloped capsids). VLPs that display high symmetry and are not lipid-enveloped tend to be more suited for bioengineering purposes; as such, protein complexes are more amenable toward atomic-scale structural elucidation. Thus, the effects of chemical and biological modifications, be it on the interior or exterior of the VLP, can be better predicted and understood [55].

The cowpea chlorotic mottle virus (CCMV) is a plant-derived icosahedral virus, consisting of 180 copies of capsid protein (Figure 2). By chemically modifying glucose oxidase (GOX) and gluconokinase (GCK) with DNA tags, Cornelissen and co-workers were able to encapsulate these enzymes within CCMV to craft a nanoreactor containing an enzyme cascade, in which glucose was oxidized to gluconic acid by GOX and then phosphorylated by GCK to form gluconate-6-phosphate [56]. The in vitro assembly of CCMV with chemically modified enzymes was made possible as the arginine rich N-terminal region of the CCMV capsid protein initiates capsid assembly in the presence of a sufficient concentration of negatively charged macromolecules, such as its cognate RNA cargo or DNA-modified proteins [57]. Approximately 1 GOX molecule and 1–2 GCK were confined within the CCMV shell. The apparent *k_cat_* values of both encapsulated enzymes were approximately twice that of free enzymes, which the authors surmised was due to improved substrate channeling in the confined system. The ability of the CCMV particle to disassemble at pH ≥ 7.5 and re-assemble at pH 5 or in the presence of negatively charged macromolecules permits robust titration of the number of cargo protein to be confined within the CCMV [57]. This property has been exploited to study enzyme mechanisms at a single-molecule level [58].

**Figure 4:**
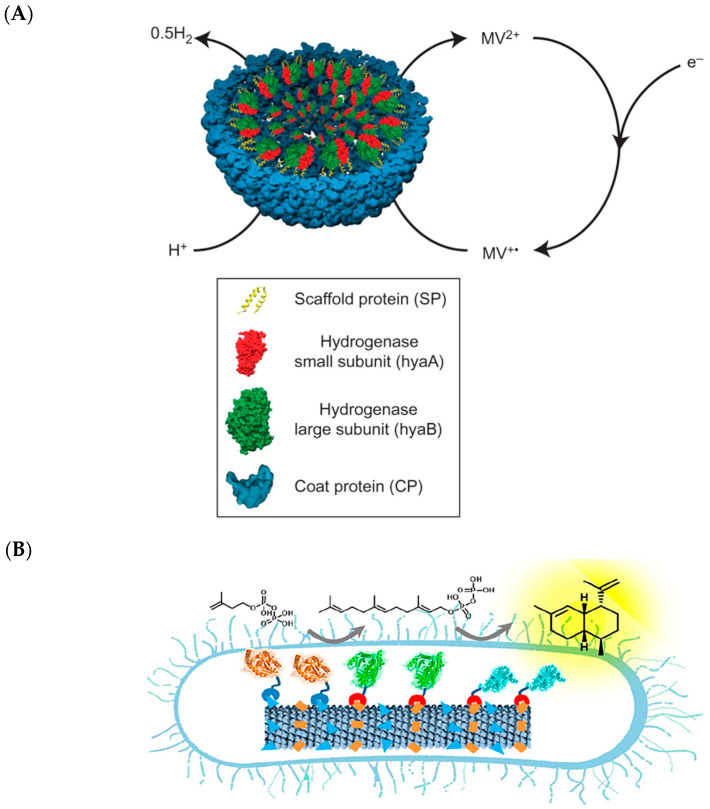
Various applications of VLPs for enzyme scaffolding. (**A**) Repurposing the P22 capsid to house the oxygen-sensitive hydrogenase complex. Reprinted from Douglas and co-workers [63]. Copyright 2016 Springer Nature. (**B**) Conjugation of three enzymes involved in terpene biosynthesis on the TMV capsid increases titers of the desired product, amorpha-4,11-diene. Reprinted from Xia and co-workers [67]. Copyright 2020 American Chemical Society.

The head portion of the P22 phage particle (Figure 2) has been extensively engineered for biotechnological utility, due to its stability and established protocols for recombinant production [59]. This VLP is made from 420 copies of a capsid protein that, with the assistance of luminal scaffolding proteins, assemble into a *T* = 7 icosahedron measuring approximately 58 nm in diameter [60]. P22 VLPs are also thermostable, retaining the capsid structure at 75 °C for at least 20 min. Douglas’ group has shown that fusion of the C-terminal residues of the scaffolding protein to fluorescent proteins and foreign enzymes resulted in a large number (~80–300 molecules) of cargo contained within the P22 VLP cage [60,61,62]. By concentrating an alcohol dehydrogenase within the shell to approximately 7 mM, Douglas’ group overcame substrate inhibition present in the free enzyme while maintaining its catalytic efficiency [60]. Encapsulation of a glycosidase, CelB, within the P22 shell also allowed it to retain its activity when immobilized in a polyacrylamide gel matrix, even after the gel was dehydrated and rehydrated [62]. The P22 container has also been repurposed for housing multi-enzyme biosynthetic reactions. It was used to sequester a [NiFe]-hydrogenase complex, conferring resistance against proteolytic cleavage and inactivation by heat and oxygen (Figure 4A) [63]. The ability to protect the hydrogenase complex from oxygen inactivation is of great significance, as hydrogenases are generally sensitive to inhibitory occupation of the metal cluster active site by O_2_ [64]. Under the same ambient atmospheric condition, encapsidation of the hydrogenase complex in the P22 capsid resulted in an approximately 100-fold increase in activity compared to the free enzyme. More recently, the P22 capsid has been used to house two enzymes involved in the biosynthesis of glutathione from its constituent amino acids—glutamic acid, cysteine, and glycine [65]. Shells containing these enzymes were applied to HEK293 cells and produced glutathione in situ, protecting the cells against induced oxidative stress. It can be envisioned that modifying the shell exterior with appropriate cellular targeting motifs would further improve its utility for translational therapeutic applications.

The biosynthesis of many natural products involves multiple enzymes that are often localized together within the native producer [66]. Subcellular regions where biosynthetic enzymes conglomerate are termed metabolons. Recapitulation of multi-enzyme pathways in heterologous hosts often gives significantly lower titers of the desired product compared to the native producer. A commonly cited reason is inefficient substrate channeling between the biosynthetic enzymes in the foreign host [11]. The tobacco mosaic virus (TMV) (Figure 2) is a homomeric VLP that resembles an alpha-helix [39]. The TMV shell has been tailored to serve as a synthetic metabolon for three enzymes involved in the synthesis of amorpha-4,11-diene (Figure 4B), a sesquiterpene that is the precursor to the anti-malarial drug artemisinin [67]. To localize the enzymes to the TMV scaffold, Xia and co-workers utilized the SpyCatcher/SpyTag and SnoopCatcher/SnoopTag protein conjugation domains/sequences that were genetically fused to the TMV scaffold and the enzymes [67]. The smaller SpyTag and SnoopTag sequences were installed on the C-terminus of the TMV protomer, while the larger SpyCatcher and SnoopCatcher protein domains were fused to the enzymes. They demonstrated that while free enzymes did not produce detectable levels of amorapha-4,11-diene, likely due to the inherently low intracellular levels of the isopentenyl pyrophosphate precursor, the scaffolded pathway could produce the target molecule. Overall, the VLP platform offers solutions to longstanding issues encountered in engineering biosynthetic reactions, namely poor metabolic flux, enzyme instability, and suboptimal enzyme activity.

### 2.2. Encapsulins, Ferritins, and Small Heat Shock Proteins

**Figure 5:**
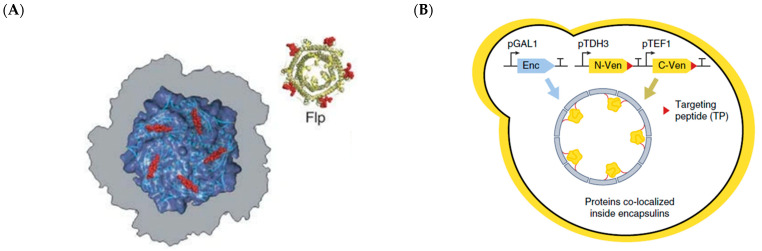

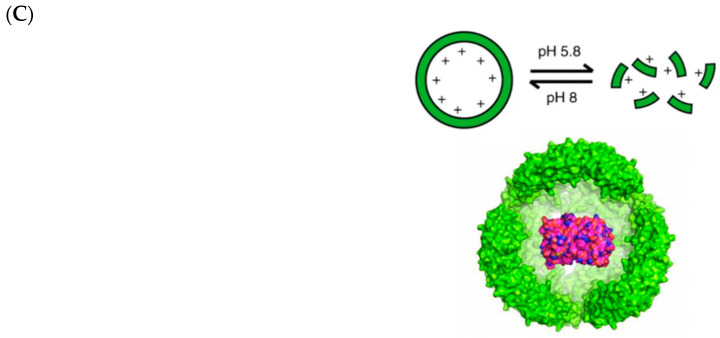
Nanoscale protein compartments from bacteria and archaea can be engineered to house enzymes. (**A**) Structural model of how cargo proteins are encapsulated by the *T. maritama* encapsulin shell. The C-terminal EP sequence (colored red) of the Flp protein docks into a hydrophobic pocket on the interior of the shell. Reprinted from Ban and co-workers [69]. (**B**) Assembly of *A. fulgidus* ferritin shells were engineered to be pH-responsive, allowing easy encapsulation of guest proteins (colored red/blue) in vitro. Reprinted from Drum and co-workers [15]. (**C**) The *M. xanthus* encapsulin shell was produced in *S. cerevisiae* and was capable of co-localizing split proteins. Reprinted from Silver and co-workers [51]. Copyright (**A**) 2008, (**B**) 2017, and (**C**) 2018 Nature Research.

Many bacterial and archaeal species that live in challenging environments express protein shells that ameliorate oxidative stress, sequester minerals, or assist in protein folding. These nanoscale protein shells (approximately 10 to 30 nm in diameter), which are typically homomeric in composition, fulfil diverse physiological functions in their hosts. Encapsulin shells are found in 2 archaeal and 15 bacterial phyla, and are postulated to act as iron storage containers and to mediate redox processes, insulating the bulk intracellular environment from reactive radical species that emerge during such processes [68]. At least two distinct morphologies have been reported for encapsulin shells. The smaller *T* = 1 icosahedral shell, such as the one from *Thermotoga maritima*, comprises 60 pentameric protomers and is 24 nm in diameter, while the larger *T =* 3 icosahedral shells, such as those from *Pyrococcus furiosus* and *Myxococcus xanthus* (Figure 2), comprise 180 protomers and are about 31 nm in diameter [31]. Encapsulin shells contain pores measuring about 6 Å in diameter, allowing free diffusion of some small molecules [31]. Guest proteins can be targeted into the shell using a conserved C-terminal sequence that interacts with a hydrophobic pocket in the shell lumen (Figure 5A) [69]. The robust nature of encapsulin shell assembly and of shell luminal targeting has prompted the engineering of encapsulin shells as bespoke nanoreactors.

A *T* = 1 encapsulin shell from *Mycolicibacterium hassiacum*, a thermophilic mycobacterium, has been engineered to separately house four different enzymes—a bacterial peroxidase, a laccase, a catalase, and a flavoenzyme [18]. In spite of each of the abovementioned cargo enzymes having distinctively different molecular sizes, oligomerization states, cofactor requirements, and catalytic mechanisms, all the enzymes were successfully targeted within the shell. Encapsulation had varying effects on the catalytic efficiencies of the enzymes, underscoring the need to determine the effects of enzyme scaffolding on a case-by-case basis. Nonetheless, the *M. hassiacum* encapsulin shell appeared to confer thermostability on the heat sensitive peroxidase and flavoenzymes. The relatively straightforward assembly of the encapsulin shell facilitates its recombinant production in eukaryotic cells. The *M. xanthus* encapsulin was expressed in the budding yeast, *Saccharomyces cerevisiae*, to serve as an orthogonal organelle in this widely used eukaryotic cell factory [51]. By fusing encapsulation peptides (EPs) to the two splits parts of a yellow fluorescent protein (YFP), the shell was able to co-localize the two parts and restore fluorescence (Figure 5C). As a proof-of-concept that biosynthesis could take place inside these shells, an in vitro assay was carried out whereby the benzylisoquinoline alkaloid (BIA), norcoclaurine, was synthesized via a Pictet-Spengler reaction from 4-hydroxyphenylacetaldehyde (4-HPAA) and dopamine. The encapsulated decarboxylase enzyme, Aro10p, catalyzed the in situ production of 4-HPAA from 4-hydorxyphenylpyruvate. There has been a strong impetus to utilize yeast platforms for manufacturing diverse plant alkaloids, and recognition that aldehyde intermediates such as 4-HPAA are channeled away by the yeast’s endogenous enzymes [2,20,70]. The establishment of fully orthogonal intracellular compartments in yeast should help overcome the problem of byproduct generation in natural product synthesis.

The *M. xanthus* encapsulin has also been produced in HEK293 cells for encapsulating cargo that can serve as imaging or detection agents in mammalian cells [71]. Westmeyer and co-workers targeted the tyrosinase enzyme inside the shell to limit the toxic effects of melanin on the cell. Melanin has recently been reported as a photo-absorber for use in optoacoustic tomography [72]. This molecule is typically contained within lipid-enclosed organelles termed melanosomes, but endogenous organelles are difficult to engineer without significantly affecting host physiology. Tyrosinase-containing encapsulin shells essentially functioned as synthetic melanosomes that limited the effect of melanin on cell viability. As further demonstrations of the broad utility of the encapsulin shell for bioimaging, the reporter enzymes ascorbate peroxidase APEX2 and cystathionine γ-lyase were also targeted within the shell and generated the appropriate signals [73,74]. This study sets the stage for using encapsulin nanoshells for exerting fine spatial control of enzymes in cellular imaging.

Ferritin shells are highly conserved in all three domains of life and function as iron storage containers, sequestering approximately 2000 iron atoms in their core, as part of cellular iron homeostasis [75]. The shell, which has a diameter of 12 nm, is formed using 24 protomers that assemble with an overall octahedral symmetry. The shells also show intrinsic ferroxidase activity, oxidizing Fe^2+^ to Fe^3+^ using O_2_ as the electron acceptor [76]. Ferritins originating from extremophiles are exceptionally stable to heat, chemical denaturants, and ionizing radiation [77]. The ferritin cage from the archaeon *Archaeoglobus fulgidus* (Figure 2) has been utilized to host an engineered GFP with 36 positive charges, GFP(+36) [78]. This cargo was chosen as the interior of the ferritin cage has high affinity for positively charged species, and indeed, approximately five GFP(+36) molecules were encapsulated, corresponding to 70% of the theoretical maximum packing capacity within this protein cage. Hilvert and Tetter then used GFP(+36) to mediate encapsulation of three enzymes with disparate substrate profiles, carbonic anhydrase, retro-aldolase, and Kemp eliminase. Around two to three copies of the fusion proteins were encapsulated per shell, due to the increase in overall mass of the fusion cargo. The shells conferred resistance against proteolytic and organic solvent-induced degradation on the enzymes, and in the case of retro-aldolase, increased its T_m_ from 60 °C to 70 °C. In a similar vein, Drum and co-workers utilized the *A. fulgidus* ferritin shell to stabilize several classical reporter enzymes against thermal and biochemical denaturation [15]. A key limitation in utilizing ferritin shells is paradoxically due to their robustness: harsh conditions are needed to disassemble these shells and such conditions would almost invariably damage the encapsulated biomolecules [79]. The authors overcame this problem by mutating one of the residues in the shell protomer interface to histidine to make a pH-titratable switch that controls shell assembly/disassembly. Below pH 5.8, the interfacial side chain of histidine is predominantly protonated and the protomers electrostatically repel each other, resulting in shell disassembly (Figure 5B). At pH 8, the histidine side chain is largely deprotonated, and thus, the shell reassembles. The more pH-responsive shell is, therefore, more suited for applications where physiological conditions need to be adhered to. Heddle and co-workers extended the utility of the enzyme-loaded ferritin shells into nanofabrication by incorporating *T. maritima* ferritin shells into gold nanoparticles (AuNP) lattices [80]. The encapsulated lysozyme experienced a two-fold decrease in activity, likely due to reduced access to substrate within the lattice. Nevertheless, the ability to introduce enzymes into nanofabrication technologies, via encapsulation within robust ferritin shells, opens up avenues for their use in domains that are typically limited to inorganic materials.

Small heat shock proteins (sHSP) function to prevent nascent protein chains from misfolding and to assist in refolding aggregated proteins [81]. Many sHSPs form oligomeric complexes, consisting of 2 to 32 subunits, that are important for their chaperone activity. The *Methanococcus jannaschii* Hsp16.5 has been structurally characterized (Figure 2); 24 protomers assemble into a cage with an overall octahedral symmetry, with an external diameter of 12 nm and 3 nm pores [75]. The *M. jannaschii* Hsp16.5 is stable between pH 5 to 11 and up to 70 °C, which is not unexpected since the native host is a thermophile [82]. Thus far, this shell has been used to prevent aggregation of the heat-labile citrate synthase [83], but no other reports of its use in tandem with non-cognate enzymes are known. Given the thermostability of the *M. jannaschii* Hsp16.5 shell and its ability to stabilize proteins against denaturation, this shell has the potential to be a platform for improving enzyme stability.

### 2.3. Bacterial Microcompartments

**Figure 6:**
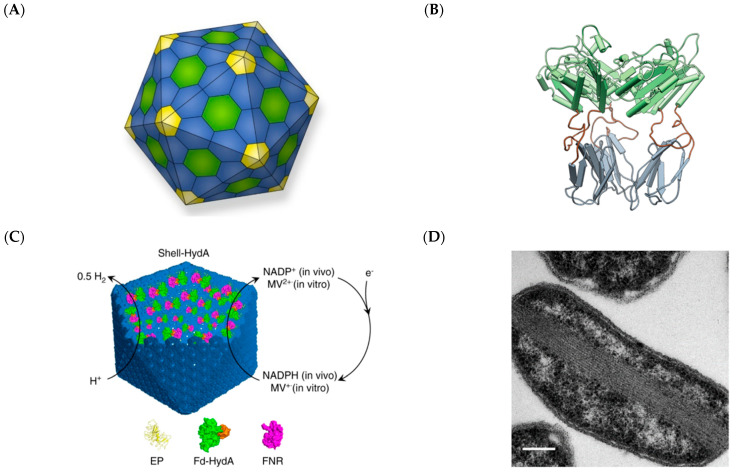
Repurposing BMCs and BMC components into scaffolds for enzyme catalysis. (**A**) Model of the HO-BMC, with BMC-H colored blue, BMC-P yellow, and BMC-T green. Reprinted from Kerfeld and Kirst [97]. Copyright 2019 Springer Nature. (**B**) Grafting of the SpyCatcher (below, grey) domain into a loop region within the HO-BMC-T protein (top, green) for covalent conjugation of protein cargo. Reprinted from Kerfeld and co-workers [54]. (**C**) The alpha-carboxysome has been repurposed for hosting the hydrogenase enzyme, HydA, and a ferredoxin-NADP(+) oxidoreductase (FNR) to produce molecular hydrogen. Reprinted from Liu and co-workers [93]. (**D**) PduA*, a variant of the PduA BMC-H protein, forms elongated rod-like structures within *E. coli*, which can be modified to host enzymes. Reprinted from Warren and co-workers [95]. Copyright (**B**) 2018, (**C**) 2020, and (**D**) 2017 Nature Research.

Most autotrophic and some heterotrophic bacterial species express bacterial microcompartments (BMCs) to carry out specialized metabolic processes [30]. BMCs are protein shells, ranging from 40 to 400 nm in diameter, and can be divided into two main classes based on their physiological roles. Shells that facilitate the fixation of inorganic carbon in the light-independent stage of photosynthesis are termed carboxysomes. These are found in virtually all cyanobacteria and certain chemoautotrophic bacteria. Shells involved in the catabolism of alcohols, amino-alcohols, and other non-conventional carbon sources are termed metabolosomes. Native BMCs shells are highly intricate, consisting of hundreds to thousands of protomers of which there are three distinct types [84]. The BMC-H shell protein assembles into hexamers that form the facets of the shell and are the stoichiometrically major components (Figure 6A). The central pore that emerges in the hexameric arrangement allows diffusion of small metabolites across the shell. The BMC-P shell protein assembles into pentamers that cap the vertices of the shells and are a stoichiometrically minor component. The BMC-T shell protein, which contains a tandem repeat of the BMC-H domain, assembles into trimers that form a central pore, akin to the BMC-H proteins. However, the central pores of BMC-T shell proteins can be dynamically gated in response to changing concentrations of key metabolites, and when fully open, they are considerably larger than the constitutively open pores in BMC-H hexamers, allowing influx of larger molecules such at ATP [85]. While BMC shell architecture is more complicated in relation to other homomeric prokaryotic protein shells, the heteromeric nature of BMC shells presents avenues for multivalent enzyme scaffolding that may not be as feasible in homomeric shells.

Initial efforts in repurposing BMCs involved the use of all or almost all of the shell-encoding genes in the native BMC operon, due to the difficulty in deconvoluting the exact role and essentiality of each component in the wild-type shell [86,87,88]. Warren’s group recapitulated the PDU (propanediol utilization) metabolosome in *E. coli* by expressing seven shell proteins—PduABJKNUT [86]. The natural capability of the PDU metabolosome to sequester propionaldehyde, a volatile and reactive metabolite, encouraged the refashioning of the shell to sequester acetaldehyde, an intermediate in the enzymatic reduction of pyruvate to ethanol [52]. They identified that N-terminal sequences of two PDU luminal enzymes (PduP and PduD) could serve as EPs to mediate encapsulation of non-cognate cargo. By co-expressing the shell proteins, PduP^1–18^-PDC (first 18 residues of PduP fused to pyruvate decarboxylate) and PduD^1–18^-ADH (first 18 residues of PduD fused to alcohol dehydrogenase), Warren and co-workers were able to construct an ethanol nanoreactor within *E. coli*. The strain expressing EP tagged enzymes with shells had approximately 50% higher titers of ethanol than the strain expressing shells but with untagged enzymes. The group built on this work to engineer the PDU shell for synthesis of 1,2-propanediol [89]. This was done by installing the PduP^1–18^ and PduD^1–18^ EP sequences to four enzymes that sequentially converted glycerol to 1,2-propanediol. Surprisingly, the strain expressing EP-tagged enzymes but without PDU shell co-expression produced around 40% more 1,2-propanediol than the strain expressing both tagged enzymes and shell. The strain expressing EP-tagged enzymes only also produced about 4.5 times more 1,2-propanediol than untagged enzymes. This led the authors to conclude that the EP tags induced aggregation in the enzymes, leading to confinement within inclusion bodies in *E. coli*. Nevertheless, the enzymes remained active and the higher metabolic flux due to enzyme co-localization led to overall higher titers. These studies demonstrate that the use of protein shells for enzyme scaffolding may not necessarily improve the overall effectuality of the enzyme(s), and each design needs to be verified experimentally. This is due to the numerous factors involved, such as the effect of EP on enzyme, metabolic investment in expressing the shells, effectiveness of encapsulation, and activity of enzymes within the shells, among others.

Silver’s group has recapitulated the alpha-carboxysome from *Halothiobacillus neapolitanus* in *E. coli* through transplantation of the *H. neapolitanus cso* operon, which encodes 10 genes [87]. The resultant *E. coli* strain gained the ability to fix CO_2_ due to encapsulation of RuBisCO and carbonic anhydrase within the carboxysome shell. RuBisCO is a catalytically inefficient enzyme that can be inhibited by O_2_, preventing it from binding to CO_2_ [90]. The carboxysome shell increases the efficiency of RuBisCO by blocking ingress of O_2_ while promoting the entry of HCO_3_^−^, which is converted to CO_2_ within the shell by the action of carbonic anhydrase. Building upon this work, Price and co-workers sought to build the carboxysome in plants as a way of improving carbon fixation in C_3_ plants. They replaced the tobacco plant’s endogenous RuBisCO in the chloroplasts with that from the cyanobacterium *Cyanobium*, and co-expressed two carboxysome structural proteins, CsoS1A and CsoS2 [35]. CsoS1A is a BMC-H protein while CsoS2 is a luminal scaffolding protein that is critical for the formation of the alpha-carboxysome by tethering RuBisCO with the shell proteins [91]. Shells resembling native carboxysomes formed in the plant’s chloroplasts and could support plant growth. While the transgenic plants grew poorly compared to the wild-type, and recombinant RuBisCO within the simplified carboxysomes displayed lower *k*_cat_ compared to native RuBisCO isolated directly from *Cyanobium*, this study demonstrates that useful structural elements from ostensibly complex BMCs can be used for supporting enzyme catalysis in higher organisms.

In recent years, there has been a focus on simplifying shell assembly to facilitate more widespread adoption of BMC shells in various systems. This endeavor has been actuated by the structural elucidation, at near-atomic resolution, of a BMC shell from the myxobacterium *Haliangium ochraceum* (HO-BMC) by Kerfeld’s group (Figure 2) [41]. The HO-BMC shell is a *T* = 7 icosahedron that is 40 nm in diameter and consists of 60 BMC-P, 360 BMC-H, and 60 BMC-T protomers. While the HO-BMC is smaller and simpler than many other known BMC systems, the regular geometry and atomic-level details of its structure has accelerated higher precision configuring of BMC shells as enzyme scaffolds. Kerfeld’s group has grafted the SpyCatcher domain into a luminal loop region of the HO-BMC-T protein to mediate covalent conjugation of SpyTagged proteins (Figure 6B) [54]. They have also circularly permuted structural elements within the HO-BMC-H protein such that its amino and carboxy ends are facing into the shell [92]. This has allowed protein cargo to be loaded into the HO-BMC by direct fusion to either ends of the permuted HO-BMC-H. In both cases, the modified protomers did not significantly perturb the overall structure of the HO-BMC. Liu and co-workers recently reported a simplified *H. neapolitanus* alpha-carboxysome, consisting of six shell proteins (CsoS1ABCD and CsoS4AB) and CsoS2, that served as a hydrogen-producing nanoreactor (Figure 6C) [93]. They encapsulated the *Chlamydomonas reinhardtii* [FeFe]-hydrogenases and ferredoxin, along with *E. coli* ferredoxin oxidoreductase into the simplified alpha-carboxysome by fusing these enzymes to CsoS2 C-terminal sequences. Though [FeFe]-hydrogenases are highly sensitive to oxygen, even more so than [NiFe]-hydrogenases [94], the strain co-expressing the hydrogenase complex with the shell produced approximately three times more H_2_ compared to the strain expressing just free enzymes, under the same aerobic culture conditions. These studies showcase prospects in utilizing BMCs for directed loading of enzymatic cargo and for improving the function of enzymes that are highly sensitive to their microenvironment.

Some individual BMC shell components have been found to be able to support enzyme catalysis even when not incorporated as part of a shell. Many BMC-H type proteins, in the absence of other shell components, have a strong propensity to self-assemble at high concentrations into nanoscale structures, such as rods, rosettes, or extended sheets [84]. Warren’s group created PduA*, a variant of the BMC-H protein PduA, through a serendipitous discovery. PduA* forms filaments that span across the *E. coli* cytoplasm (Figure 6D) [95]. They utilized acid-base coiled-coil interactions to target the enzymes ADH and PDC to the PduA* synthetic cytoskeleton and found that the strain with scaffolded enzymes produced approximately two-fold ethanol compared to the strain expressing just the enzymes. Another BMC-H type protein, EutM, has also been utilized as an enzyme scaffold [96]. The SpyCatcher domain was installed on 12 EutM orthologs and the ADH enzyme was fused to SpyTag. The resulting scaffold-enzyme conjugates displayed various morphologies under TEM, with some appearing as fibrils and some as films. The EuM scaffolds also had differing effects on the enzyme activity, which the authors credited to the pH microenvironment created within the various EutM orthologs. Nonetheless, most of the scaffolds conferred stability to the enzyme over a 48-h incubation period under ambient conditions compared to the free enzyme.

### 2.4. Cellulosomes

Cellulosomes are multienzyme complexes, produced extracellularly by some anaerobic cellulolytic bacteria species, that break down lignocellulosic plant material into smaller soluble molecules that can be consumed by the bacteria [98]. Cellulosomes serve to organize cellulolytic enzymes and are either attached to the bacterial cell wall or secreted into solution. The multicomponent scaffoldin protein serves as the backbone of the cellulsome; on it are carbohydrate binding modules (CBM) and cohesin modules that serve as docking sites for enzymes. The cellulolytic enzymes contain a dockerin domain that binds to the cohesin modules with high affinity (*K*_D_~10^−9^–10^−12^ M) in the presence of Ca^2+^ [25]. The cellulosome has evolved for optimizing cascade enzymatic reactions [98]. The CBM increases proximity of substrate to the enzymes, while docking of the various cellulolytic enzymes, each with their own substrate specialty in the degradation of chemically complex and recalcitrant lignocellulosic material, on the same scaffold, improves enzyme synergism. Despite its intricacy, the modularity of the cellulosome scaffold has garnered attention for accommodating cascade reactions.

**Figure 7:**
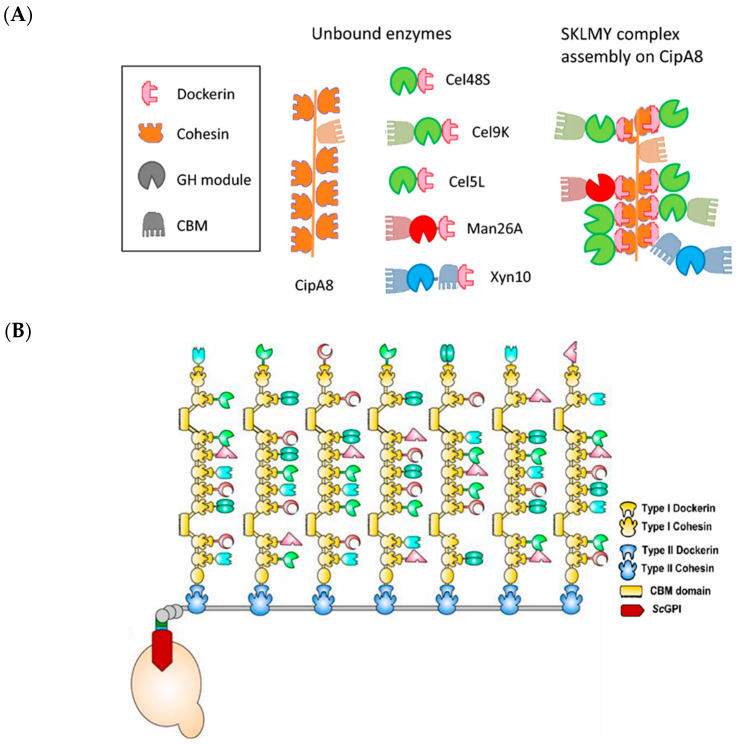
Synthetic cellulosomes can serve as scaffolds for optimizing cascade enzymatic reactions. (**A**) A synthetic cellulosome, with CipA8 as a scaffold, was formed by mixing cellulases (Cel48S, Cel9K, Cel5L), mannase (Man26A), and xylanase (Xyn10) in stoichiometric ratios. Reprinted from Zverlov and co-workers [99]. Copyright 2018 Springer Nature. (**B**) A cellulosome that can host 63 enzymes was displayed on the cell surface of *K. marxianus* through anchoring to the protein glycosylphosphatidylinositol from *S. cerevisiae* (*Sc*GPI). Enzymes are depicted as the various green, cyan, and red shapes. Reprinted from Li and co-workers [53]. Copyright 2020 National Academy of Sciences (USA).

Zverlov and co-workers have created a synthetic cellulosome with the aim of quickly tuning and optimizing the enzymatic features and activities for various cellulosic substrates [99]. They based their synthetic cellulosome on a thermostable variant from *Clostridium thermocellum*. The authors attempted to recombinantly express all 73 putative dockerin-containing cellulosomal proteins in *C. thermocellum* and identified the glycoside hydrolases (GH) Cel48S, Cel9K, and Cel5L as the minimal components required for digestion of softwood pulp. They then combinatorially screened for mannases and xylanses to supplement the aforementioned enzymes and found Man26A and Xyn10 to have increased the overall activities of the synthetic cellulosome (Figure 7A). This minimized cellulosome reached 60% of the activity of commercial cellulosome cocktail, which contains significantly more components, many of which are not well characterized [100]. This study sets the stage for further optimizing cellulosome function from known parts in a mixed semi-rational/combinatorial screening methodology.

Genetically tractable yeast species, such as *S. cerevisiae*, *Pichia pastoris*, and *Kluyveromyces marxianus*, offer a number of established tools for cell surface display of protein complexes [101]. Consequently, yeast has been a relatively popular platform for cellulosome engineering, especially in the view that the anaerobic bacteria from which these protein complexes are derived can be challenging to culture and manipulate [102]. A mini-cellulosome displaying three orthogonal cohesion domains was expressed in *S. cerevisiae* for attachment to three enzymes—ADH, formaldehyde dehydrogenase, and formate dehydrogenase fused with the partner dockerin domains [103]. This linear pathway oxidized methanol to CO_2_, generating NADH for the yeast host. It was found that the strain with scaffolded enzymes had a five-fold increase in NADH productivity than with free enzymes. *Pichia pastoris*, an emerging yeast cell factory, has been recently engineered to display a mini-cellulosome to convert cellulose to ethanol [104]. To extend the use of the cellulosome scaffold for more complex enzyme pathways, Li and co-workers have engineered a cellulosome in *K. marxianus* capable of housing up to 63 enzymes (Figure 7B) [53]. The ordered nature of the cellulosome improved metabolic flux between enzymes, and its modular nature facilitated introduction of accessory enzymes, such as laccases, that assisted in cellulose degradation. Overall, the yeast host expressing this large cellulosome achieved 8.6 g/L of ethanol from cellulose, which was the highest titer reported thus far for synthetic cellulosomes at the time of writing.

### 2.5. Hydrophobins

**Figure 8:**
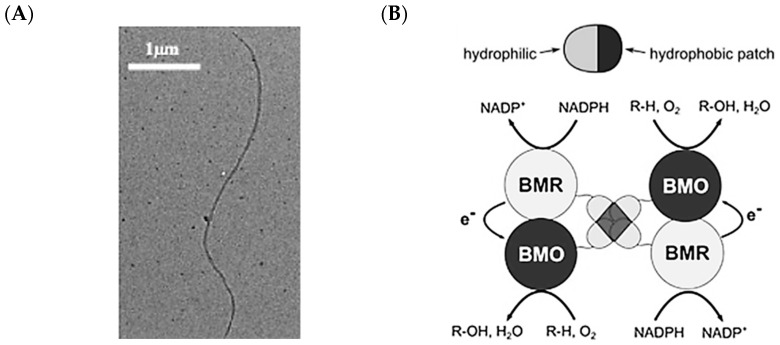
Hydrophobins as a novel platform for enzyme immobilization. (**A**) Vmh2 forms fibrils in solution. Reprinted from Giardina and co-workers [108]. Copyright 2017 Wiley Publications. (**B**) The HFBI hydrophobin brings CYPP450 reductase (BMC) and monooxygenase (BMO) domains in close proximity to each other, facilitating electron exchange. Reprinted from Urlacher and co-workers [110]. Copyright 2016 Frontiers Media.

Hydrophobins are small (<15 kDa) proteins, produced by filamentous fungi, that are characterized by eight conserved cysteine residues that form four disulfide bridges [105]. These proteins have both hydrophobic and hydrophilic patches on their surfaces (Figure 2), leading them to strongly cluster in solution and on surfaces due to the strong hydrophobic effect present [106]. Hydrophobins are classified into two groups based on sequence similarity and biophysical properties. Class I hydrophobins form rodlet-like structures resembling amyloid fibrils (Figure 8A) and remain insoluble even in boiling sodium dodecylsulfate (SDS), while class II hydrophobins are soluble in SDS [107]. Hydrophobins can be an attractive platform for enzyme immobilization in food and pharmaceutical applications, as some variants are produced by generally regarded as safe (GRAS) fungi that are consumed by humans.

The class I hydrophobin from *Pleurotus ostreatus* (oyster mushroom), Vmh2, has been utilized for immobilization of enzymes in biosensing applications. Giardina’s group fused glutathione S-transferase (GST) to Vmh2, which served to increase the solubility of the hydrophobin and also to act as a biosensor for the detection of the pesticides, molinate, and captan [108]. GST catalyzes the electrophilic substitution of glutathione (GSH) to 1-chloro-2,4-dinitrobenzene (CDNB), forming a yellow CDNB-GSH adduct. Both molinate and captan inhibit GST activity, and hence, decrease in GST activity, as determined by the CDNB-GSH adduct, which correlates with increasing concentrations of the pesticides. A key advantage of the Vmh2-GST fusion was that the biosensor enzyme could be easily immobilized on commonly used polystyrene 96-well plates, facilitating prolonged or multiple uses of the enzyme (up to 60 days or 50 cycles). Fusion of Vmh2 to GST also increased the sensitivity of the enzyme, decreasing the limit of detection (LOD) by one order of magnitude for molinate and two orders for captan compared to previous work. Another class I hydrophobin, Ccg2 from *Neurospora crassa* (red bread mold), was similarly used to improve enzymatic detection of another pesticide, glyphosate [109]. In this case, the sensor enzyme was 5-enolpyruvylshikimate-3-phosphate (EPSPS), which catalyzes the reaction between phosphoenolpyruvate and 3-phosphoshikimate, with the release of inorganic phosphate. This reaction is inhibited by glyphosate. While EPSPS is not commonly reported as a fusion protein partner to increase the solubility of partner proteins, in this case it was found that fusion to EPSPS was necessary to solubilize Ccg2. The sensitivity of the EPSPS-Ccg2 assay for inorganic phosphate detection was comparable to commercial ELISA kits. The key advantage of the developed assay was its applicability for on-site testing with simple instrumentation.

A foray in exploiting hydrophobins for multi-enzyme catalysis was to utilize HFBI (Hydrophobin I) from *Trichoderma reesei* for improving the catalytic efficiency of a cytochrome P450 (CYP450) system [110]. The catalytic efficiency of CYP450 systems depends on efficient electron transfer between the heme-containing monooxygenase enzyme and its redox partner, which transfers electrons from NAD(P)H to the monooxygenase. Heterologously expressed CYP450 systems often suffer from diminished efficiency due to loss of co-localization between the monooxygenase and reducing partner [111]. As proof-of-concept that hydrophobins could assist the electron transfer process in CYP450 systems, Urlacher and co-workers split the *Bacillus megaterium* BM3, a special CYP450 in which both monooxygenase and reductase domains are found as a single protein chain, into its parts—BMO (monooxygenase) and BMR (reductase) (Figure 8B). When fused to HFBI, both BMR and BMO were found to be soluble and the electron coupling efficiency of the BMO–HFBI and BMR–HFBI pairs reached 93% of that of native BM3. This was credited to the strong tendency of HFBI to aggregate with each other through the hydrophobic effect. The HFBI fused BMO and BMR also achieved about three times higher *k*_cat_ than free BMO and BMR when tested using hydroxylation of myristic acid.

While hydrophobins are robust to chemical and physical aggravation, a key limitation of using these emerging protein scaffolds is that many known hydrophobins, but not all, are insoluble, especially if recombinantly produced in *E. coli* [107]. Three main methods of overcoming this issue are (i) to fuse the hydrophobin protein to a highly water-soluble protein, such as GST, (ii) to purify the hydrophobin in denaturing conditions (e.g., 8 M urea) and to refold it by dialysis in oxidizing conditions, and (iii) to use a yeast host (e.g., *P. pastoris*), which has a protein folding machinery more analogous to the native fungal producers [108,109,110,112]. Hence, the issue of solubility is paramount in any endeavor using hydrophobins as scaffolds.

### 2.6. Vault Ribonucleoprotein Complexes

**Figure 9:**
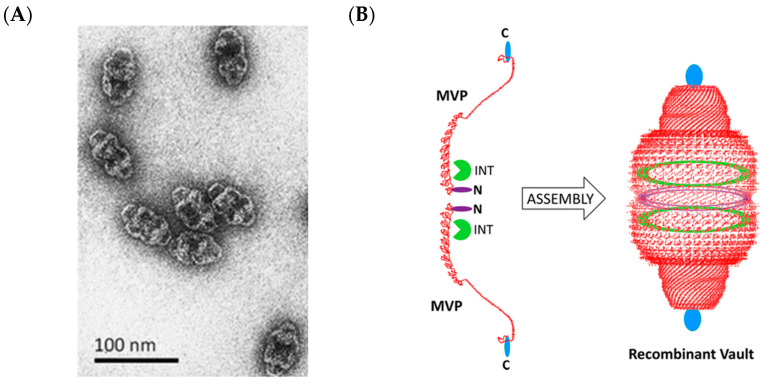
Vault complexes as an emerging eukaryotic-derived shell for biomolecular scaffolding. (**A**) TEM micrographs of vault. (**B**) Schematic view of vault, indicating the N- and C-termini along with the INT binding region. These are regions for modification of the vault shell. Reprinted from Kickhoefer and co-workers [119]. Copyright 2017 American Chemical Society.

The vault ribonucleoprotein complex, or more simply referred to as vault (Figure 2), is a highly conserved eukaryotic compartment that has a protein membrane, composed of 78 chains of the major vault protein (MVP), each of which is approximately 100 kDa in mass [113]. The distinctive arch shape of the vault (Figure 9A) resembles the architectural vaults in Roman Catholic cathedrals, hence the name. The exact cellular function(s) of the vault is currently not ascertained, though it has been found to span across the nuclear envelope and may play roles in transport of molecules across the nucleus, nuclear pore assembly, and signaling [114]. The MVP interaction domain (INT) is a 147-residue peptide that directs proteins into the vault, forming two rings above and below the middle of the shell [115]. Due to their low immunogenicity and relative ease of cargo loading, vault has been used for packaging of macromolecular and small molecule therapeutics.

Reports on the use of vault for supporting enzyme catalysis are currently sparse. The widely used reporter enzyme luciferase could be packaged within the vault via fusion to INT, and its activity was retained [116]. Using a similar methodology, manganese peroxidase (MnP) was also encapsulated by vault. MnP is a fungal enzyme that oxidizes Mn^2+^ to Mn^3+^ using H_2_O_2_ as the oxidizer. Mn^3+^, which is stabilized by chelation to organic acids produced by the fungal producer, proceeds to oxidize a wide range of organic compounds [117]. MnP has been used in bioremediation due to its ability to oxidize polycyclic aromatic hydrocarbons and phenolics [118]. It was found that encapsulation of MnP within vault increased the enzyme’s thermal stability (up to 40 °C), longevity in continuous catalysis (up to 24 h) in wastewater, and resistance to non-physiological pH (down to pH 3). These properties are beneficial for improving MnP’s economic viability in remediation of contaminated water.

Vault is one of the largest homomeric protein shells known, is mostly hollow, and has shown to be acquiescent in housing a plethora of cargo with diverse chemistries. Along with its low immunogenicity and the fact that it is eukaryotic in origin, there could be untapped potential in utilizing vault for hosting reporter enzymes for targeted cellular imaging/theranostics, as has been done for the encapsulin shell (see Section 2.2).

### 2.7. Lumazine Synthase Assemblies

**Figure 10:**
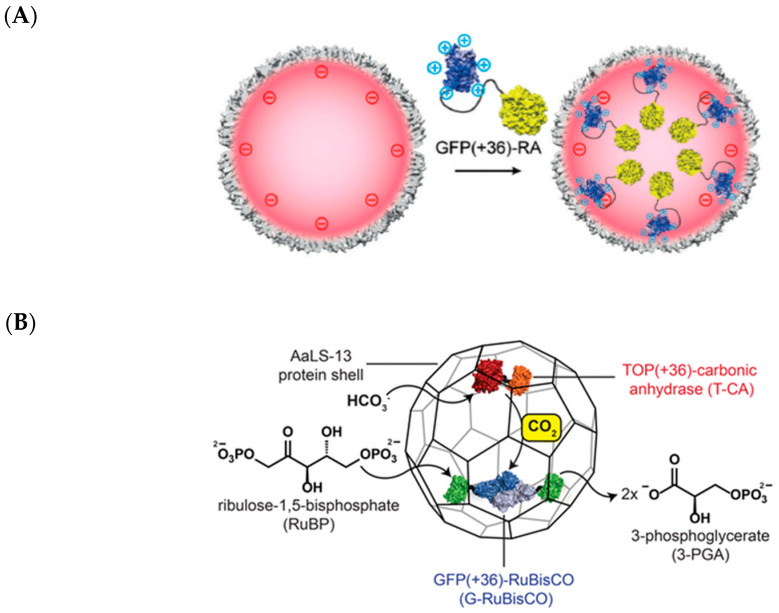
The AaLS-13 shell as a designer nanocage for hosting foreign enzymes. (**A**) Fusion of retro-aldolase (RA) to GFP(+36) allows encapsulation of the enzyme by AaLS-13. Reprinted from Hilvert and co-workers [121]. Copyright 2018 Royal Society of Chemistry. (**B**) Repurposing of AaLS-13 into a carboxysome mimic. Reprinted from Hilvert and co-workers [125]. Copyright 2016 American Chemical Society.

Lumazine synthase catalyzes a step in riboflavin (vitamin B2) synthesis and is found in bacteria, fungi, and archaea [120]. A number of quaternary arrangements of lumazine synthase have been observed, with variations dependent on the organism in which they are found [121]. Known structures of lumazine synthase include pentamers in *S. cerevisiae*, dimer of pentamers in *Brucella abortus*, and capsid-like dodecahedrons in *Aquifex aeolicus* (Figure 2). Studies done on the *Bacillus subtilis* lumazine synthase have shown that its dodecahedral lumazine synthase complex encapsulates riboflavin synthase, increasing the overall reaction rate in riboflavin biosynthesis due to local substrate enrichment [120]. As a major initial step in improving the lumazine synthase shell for hosting enzyme reactions unrelated to its native function, Hilvert’s group performed directed evolution on the *A. aeolicus* lumazine synthase (AaLS) to increase its cargo loading capacity [122]. They tagged HIV protease, which is toxic to the *E. coli* host, with 10 arginine residues such that it had a high net positive charge. The cargo was then co-expressed with a mutant library of AaLS to select for shell variants that efficiently contained the toxic enzyme within the negatively charged lumen of the shell. After four rounds of selection, a variant (AaLS-13) emerged that could encapsulate 14 copies of HIV protease, about 10-fold higher than the initial AaLS template. AaLS-13, which has an exterior diameter of 36 nm, was also later shown to be capable of capturing around 70 copies of GFP(+36). Protein cargo fused to GFP(+36) could also be efficiently targeted into AaLS-13 (Figure 10A).

The high cargo loading capacity and exceptional thermostability of the AaLS (T_m_ of wild-type ~119 °C) has made it a generalizable container for improving enzyme function [43]. AaLS-13 has been used to host a variety of enzymes, each with distinct reaction chemistries and properties: a retro-aldolase, a Kemp eliminase, a β-lactamase, a cycloamine oxidase, a monoamine oxidase, a catalase, an NADH oxidase, an aldehyde dehydrogenase, and a peroxidase [123,124]. AaLS-13 has also been used to simultaneously host two separate enzymes—RuBisCO and carbonic anhydrase—to form a shell that mimics the cyanobacterial carboxysome (Figure 10A) [125]. Unfortunately, encapsulation of both enzymes did not confer any kinetic advantage over free enzymes, nor did it prevent inhibition of RuBisCO activity by ambient oxygen. Nonetheless, the shell did protect the enzymes from protease degradation. These studies demonstrate that lumazine synthase shells are a facile platform for encapsulating a plethora of enzymes.

### 2.8. Membraneless Organelles

**Figure 11:**
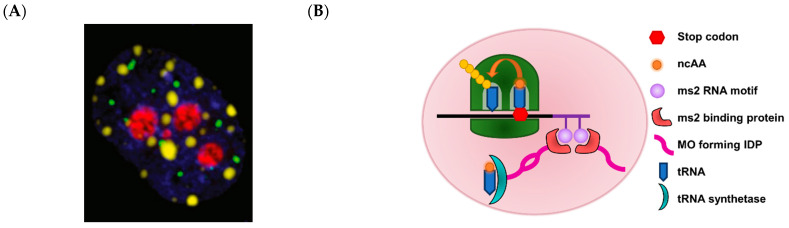
Eukaryotic MOs can be functionalized to host unrelated proteins. (**A**) Fluorescent microscopy of MOs in HeLa cells: Ddx4 (part of nuage) is fused to a yellow fluorescent protein (YFP), nucleoli colored red, and nuclear bodies colored green. Reprinted from Baldwin and co-workers [126]. Copyright 2015 Elsevier. (**B**) A synthetic MO in HEK293 cells enhanced the incorporation of the ncAA, Pyl, into proteins. Adapted from Lemke and co-workers [127]. Copyright 2019 Reinkemeier, C.D. et al.

Membraneless organelles (MO) are biomolecular condensates, composed of proteins and nucleic acids, that phase separate from the bulk intracellular aqueous environment (Figure 2) [29]. Such condensates have a dielectric permittivity more akin to polar organic solvents (e.g., DMSO) than to water. MOs tend to be amorphous in appearance and adopt a wide range of sizes. MOs are more dynamically regulated than lipid-delineated organelles, in that changes in cell physiology and extracellular environment can swiftly alter the size and abundance of MOs [126]. This dynamic regulation is concordant with findings that eukaryotic MOs, which include nuclear speckles, stress granules, nuage, and the nucleolus, are generally involved in rapid cellular processes, including stress response and cell differentiation (Figure 11A) [29]. In tandem with re-emerging appreciation of the importance of MOs with regard to cell physiology, the striking biophysical properties of MOs have garnered the attention of synthetic biologists in recent years.

A landmark study by Lemke’s group reported the formation of synthetic MOs in HEK293 cells to increase the efficiency of translational incorporation of non-canonical amino acids (ncAA) into proteins [127]. In vivo incorporation of ncAAs into proteins is often challenging as the host’s endogenous translational machinery interferes with the suppressor tRNA, leading to low suppression of stop codons that have been co-opted as ncAA codons [128]. Lemke and co-workers fused long IDPs to the suppressor tRNA synthetase and to an RNA motif (ms2) encoded in the mRNA containing the stop codon to be suppressed (Figure 11B). This strategy created a micrometer scale compartment within the cell whereby components of the ncAA translational machinery, such as the suppressor tRNA, suppressor tRNA synthetase, and target mRNA, were enriched. The MO strategy improved the selectivity of incorporation of the ncAA, pyrrolysine (Pyl) by about 10-fold. Using this platform, Pyl was incorporated not only into fluorescent proteins but also into endogenous cytosolic and membrane proteins. The use of eukaryotic MO-formation sequences (MOFS) to form protein condensates via liquid-liquid phase separation (LLPS) has been reported in *E. coli* [36]. Overexpression of spider silk and resilin proteins resulted in the formation of liquid-like compartments in *E. coli*. Enzymes involved in the conversion of L-aspartate 4-semialdhyde to 1,3-diaminopropane, an industrially useful platform chemical, were fused to these MOFS to determine if compartmentalization of these enzymes might improve the overall productivity of the simple pathway. Unfortunately, the strain with the fusion enzymes did not perform better than the strain with free enzymes. Another recent study utilized MOFS from proteins (GKAP, Shank, Homer) found to be abundant in dense regions at synapses [129]. These MOFS were utilized in two different contiguous biosynthetic pathways—menaquinone biosynthesis involving three enzymes (MenF, MenD, MenH) and farnesyl pyrophosphate biosynthesis involving two enzymes (Idi, IspA). Under in vitro conditions, the synthetic enzyme condensate improved the productivity of menaquinone production by 70% and that of farnesyl pyrophosphate by 50%. In these studies, the formation of MOs loaded with enzymes was achieved by high-level expression of the MOFS–enzyme fusion protein. The straightforward nature of this approach is a distinct advantage of using MO for enzyme scaffolding in comparison to highly structured protein shells, where the stoichiometries between the shell protomers and cargo proteins need to be titrated to produce shells optimally loaded with cargo.

A current limitation in the adoption of this emerging scaffold is that there is only preliminary data on the chemical microenvironment within MOs. Some MOs are involved in nucleic acid processing, and therefore, have been observed to concentrate nucleic acids from the surrounding aqueous environment, although basic biophysical parameters, such as pI and molecular size, are not correlated with inclusion within the MO [130]. Due to the varied molecular interactions found to contribute to LLPS in different MOFS—electrostatic, dipole, cation-π, π-π, and coiled coils, among others—the chemical selectivity of small molecule passive transport close to and within MOs need to be better understood for more tractable engineering of MO-based scaffolds [29].

## 3. Nucleic Acid Scaffolds

Nucleic acids are able to adopt a range of architectures that can serve as enzyme scaffolds for in vivo and in vitro applications. Computational design of self-assembled two- and three-dimensional nucleic acid topologies tend to yield higher predictability in comparison to protein-based scaffolds [131]. The folding of nucleic acid scaffolds into predicted structures in solution is also mostly robust, in contrast to protein scaffolds where researchers sometimes need to contend with proper protein expression and folding. On the other hand, coupling of enzymes to nucleic acid scaffolds is generally less straightforward than with protein scaffolds, and the acidic microenvironment imposed by the phosphate backbone of nucleic acids may impact the activity of pH sensitive enzymes [25,132]. This section discusses how DNA and RNA-based scaffolds can supplement enzyme function, focusing on their distinctive properties and areas of application.

### 3.1. DNA Scaffolds

**Figure 12:**
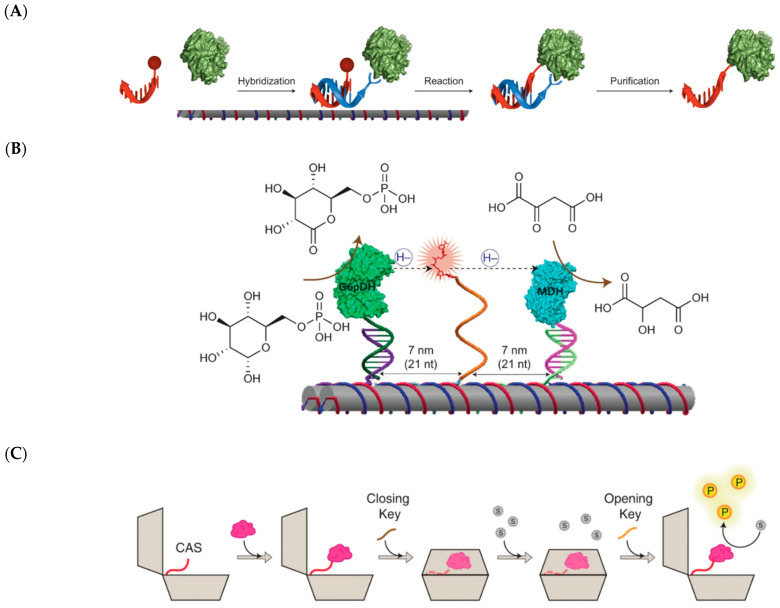
DNA can serve as sophisticated scaffolds for hosting enzymatic reactions. (**A**) Schematic of DNA-templated protein conjugation, in which a guiding strand (blue) hybridizes to the reacting strand (orange) that forms a covalent bond with the protein (green globule). Reprinted from Gothelf and co-workers [138]. (**B**) Tethering of the NADH co-factor on a flexible arm between the enzymes G6pDH and MDH on a DNA scaffold. Reprinted from Yan and co-workers [139]. (**C**) A DNA vault in which an enzyme (pink globule) is targeted within via the cargo-anchoring site (CAS), which can be closed by a “closing key” sequence. Only with the addition of an “opening key” sequence can the enzyme react with the substrate (grey circles) to generate the product (yellow circles). Reprinted from Andersen and co-workers [143]. Copyright (**A**,**B**) 2014 and (**C**) 2017 Nature Research.

DNA is arguably the most programmable of all biomolecules due to its high thermostability, predictable biophysical properties, and reliability in pairing to complementary strands [133]. This programmability, coupled with rapidly decreasing cost of DNA synthesis, has contributed to the field of DNA nanotechnology [134]. The advent of DNA origami, in which a long single stranded DNA can be folded into defined two- and three-dimensional shapes using short “staple” strands, has inspired creative use of DNA as scaffolds for an assortment of molecules [135]. Enzymes can be installed onto DNA scaffolds using non-covalent or covalent strategies. One non-covalent, high-affinity method involves expressing enzymes as streptavidin fusions and conjugating to biotin modified DNA [136]. The converse, biotinylating enzymes and attaching streptavidin to DNA, can also be done. Another non-covalent method of installation uses nitrilotriacetate (NTA) modified DNA to complex with His_6_-tagged enzymes in the presence of Ni^2+^ [137]. This strategy is convenient as the His_6_ tag can serve a second function in purification of enzymes by immobilized metal affinity chromatography (IMAC). Covalent DNA-protein attachment is less straightforward but offers undissociable conjugation. One method developed by Gothelf and co-workers, termed DNA-templated protein conjugation (DTPC), introduces the His_6_-tag followed by a single lysine onto proteins, which then binds to an NTA-modified DNA strand (Figure 12A) [138]. The NTA-modified strand hybridizes with a complementary strand carrying an aldehyde moiety, which forms a covalent bond with the aforementioned lysine via reductive amination.

The robust GOX and horseradish peroxidase (HRP) pair has been a popular proof-of-concept enzyme cascade for assessing various DNA architectures since 2008 [25]. A more recent and sophisticated example involved recruitment of the enzymes into DNA origami nanotubes with approximate diameters of 30 nm [136]. Within the nanotubes, the activities of GOX and HRP increased by approximately 5- and 3-fold, respectively. When the tubes, separately containing GOX and HRP, were dimerized, the overall activity of this simple cascade increased by 10-fold. This was attributed to improved substrate channeling within the nanotubes.

Yan and co-workers refashioned DNA to mimic flexible “swinging arms” present in multi-enzyme complexes and multifunctional mega-enzymes (e.g., polyketide synthases) [139,140,141]. They immobilized glucose-6-phosphate dehydrogenase (G6pDH) and malate dehydrogenase (MDH) on a DNA double crossover tile, in which two DNA helices placed side by side are joined by cross over of strands (Figure 12B) [142]. G6pDH oxidizes glucose-6-phosphate with the reduction of NAD^+^ to NADH, while MDH reduces oxaloacetate to malate and oxidizes NADH back to NAD^+^. The key NADH cofactor was installed between G6pDH and MDH via a poly(T)_20_ strand. The authors exploited the easy maneuverability of the DNA scaffold to find an optimal stoichiometry ratio of enzymes and NADH. The construct that gave the highest activity had a cross geometry where four MDH and four NADH were centered around one G6pDH. This scaffold gave an approximate 277-fold increase in activity over just immobilized enzymes but free cofactor. To further reinforce the advantage of substrate channeling, the authors introduced lactate dehydrogenase (LDH), which competed with MDH for NADH. They designed an assay whereby free LDH was incubated with the G6pDH-MDH scaffolds with decreasing fractions of immobilized MDH. It was determined that the activity of LDH increased linearly with decreasing fractions of immobilized MDH, signaling the importance of enzyme-cofactor proximity in the scaffold.

More recent advances in DNA origami have allowed deployment of DNA as molecular machines through dynamic alteration of their three-dimensional shape [133]. A recent and first demonstration of dynamic DNA scaffolds for hosting enzymes was the design of a DNA vault that can open and close in response to opening and closing keys (Figure 12C) [143]. The opening key binds to a lock region on the vault, causing it to open, while the closing key anneals to the opening key, removing it from the lock region. Chymotrpysin was introduced into the DNA vault by incorporating an alkynyl moiety into the cargo anchoring site (CAS), while chymotrypsin itself was reacted with azido-*N*-hydroxysuccinimide (azido-NHS) ester. These alkynyl and azido moieties react to form a 1,2,3-triazole linkage via the Huisgen cycloaddition [144]. In the presence of a fluorophore-tagged chymotrypsin substrate, it was found that the open vault exhibited approximately three times the activity of the closed vault. The authors surmised that the unexpectedly high basal activity seen in the closed vault was likely due to misfolded DNA vaults that did not properly sequester the enzyme. Nonetheless, the DNA vault created in this study can serve as a prototype for dynamically regulating enzyme activities in response to environmental signals.

### 3.2. RNA Scaffolds

**Figure 13:**
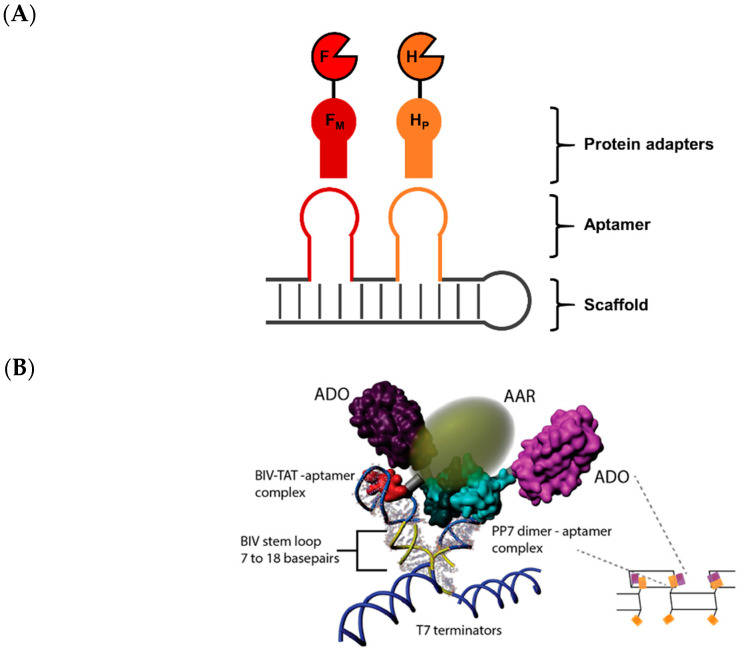
RNA aptamers are important for enzyme scaffolding. (**A**) Fusion of the aptamer binding F_M_ and H_P_ domains to the cargo proteins ferredoxin (F) and hydrogenase (H) allows these enzymes to be coupled to RNA scaffolds. Reproduced from Aldaye and co-workers [22]. (**B**) Proposed model in which the enzymes ADO and AAR are tethered to a two-dimensional RNA scaffold by aptamer binding. Reprinted from Silver and co-workers [148]. Copyright 2014 Oxford University Press.

In contrast to DNA, research in harnessing RNA scaffolds has been comparatively slow due to few established methods for binding RNA to proteins with very high affinity [145]. This is further discouraged by the predilection for RNA polymers to hydrolyze under basic conditions or by the action of RNase, a pervasive and hardy contaminant in RNA molecular work [146]. As such, the use of RNA scaffolds for enzyme tethering has been largely restricted to in vivo applications. The sizable library of RNA aptamers that bind to protein domains, mostly with moderate affinities (*K*_D_—mM–µM), has been critical for creating enzyme pathways in microbial hosts [147]. To construct an RNA scaffold capable of supporting coupled enzyme catalysis, aptamer-binding protein domains were fused to the enzymes [FeFe]-hydrogenase and ferredoxin. These enzymes work in tandem to reduce water to molecular hydrogen. The PP7 domain (H_P_) and MS2 dimeric domain (F_M_) were installed on the hydrogenase and ferredoxin, respectively (Figure 13A). Binding of the enzymes on discrete RNA hairpin duplex, one-dimensional, and two-dimensional scaffolds improved hydrogen productivity by 4-, 6-, and 24-fold, respectively, compared to free enzymes. This improvement was correlated with increased immobilization of the enzymes afforded by higher-order architectures.

Building on this work, a small collection of eight aptamer sequences and corresponding protein binders were utilized for localization of two enzymes in pentadecane production [148]. Acyl-ACP reductase (AAR) and aldehyde deformylating oxygenase (ADO) convert C_16_ acyl-ACP to the C_15_ pentadecane through an aldehyde (hexadecanal) intermediate [149]. However, hexadecanal is readily reduced by the host’s endogenous alcohol dehydrogenases to form an alcohol side product. It was reasoned that co-localization of these enzymes would reduce the extent of the side reaction. The aptamer binding peptides PP7 and BIV-TAT were installed on ADO and AAR, respectively, and co-expressed with a two-dimensional RNA scaffold (Figure 13B). The RNA scaffold offered exquisite control over the geometry and proximity between the two enzymes. A 13 to 17 bp range of hairpin stem length between the aptamer regions was tested and it was found that a distance of 14 and 16 bp offered the highest titers of pentadecane synthesis while 15 bp yielded the lowest. A model of the RNA-enzyme scaffold was proffered where at a 15 bp distance the enzymes were far from each other while at 14 and 16 bp they were in close proximity.

## 4. Molecular Tools for Constructing Enzymatic Scaffolds

An overarching theme that can be seen from the above discussion on protein and nucleic acid-based compartments and scaffolds is that expressing these compartments/scaffolds and coupling them to the enzyme(s) of interest require careful planning of the multiple steps involved. In this section, we summarize molecular tools that enable such efforts, focusing on their strengths and limitations.

### 4.1. Multipartite DNA Assembly Enabled by Synthetic Biology

The first step in co-expressing enzymes and enzymatic pathways with their target scaffold often involves piecing together numerous DNA parts—open reading frames (ORF), promoters, linkers, and terminators. The advent of synthetic biology has brought along efficient multipartite DNA assembly methodologies [150]. These developments have been leveraged by researchers seeking to design, build, and test enzyme and scaffold systems to achieve an optimal stoichiometric ratio of parts. While some researchers have made use of multiple plasmids with orthogonal selection markers for tuning expression of multiple genes, others have opted to accommodate numerous genes on a single plasmid. The key advantage of the latter strategy is that the overall metabolic burden on the host can be reduced. Three main DNA assembly methods that have found widespread success in enabling combinatorial assembly of numerous genetic parts are identified. These methodologies—BioBrick assembly, Golden Gate assembly, and Gibson assembly—have been utilized in many of the studies cited herein. The construction of BMCs and cellulosomes are discussed in relation to the DNA assembly methods, as these scaffolds have more parts compared to the others mentioned.

BioBrick assembly uses type IIP restriction enzymes (RE) recognition sites that flank the genetic part [151]. The RE recognition sites on a part are chosen such that when it is ligated to another part, the recognition sites are removed. This strategy allows genetic parts to be sequentially introduced into the destination plasmid. This “Link and Lock” BioBrick strategy was utilized to sequentially introduce seven PDU shell proteins (PduABJKNUT) into a single plasmid, allowing the contribution of each component towards shell assembly to be interrogated [86]. It was also used to construct a simplified β-cyanobacterial carboxysome, consisting of four shell proteins, by assigning ribosomal binding sites (RBS) of appropriate strengths to the components [88]. Some synthetic cellulosomes were also constructed using the BioBrick strategy [53,99,104]. Major shortcomings of the BioBrick strategy include the creation of RE ligation scars flanking each part at every stage of assembly and that digestion and ligations of parts have to be carried out sequentially.

Golden Gate assembly uses type IIS RE that cut outside of their recognition site. Ligated genetic parts can, therefore, be designed to have the RE recognition site removed after digestion. Consequently, digestion and ligation can be performed in a one-pot reaction, expediting the assembly process. Golden Gate assembly has been used in gene shuffling experiments and can be used to assembly up to 10 parts simultaneously with >90% accuracy [152]. Golden Gate assembly has been used to create synthetic compartments using five PDU shell proteins and also to create chimeric metabolosome shells [153,154]. The main limitation of Golden Gate assembly is that the RE recognition sites need to be absent from the genetic parts used.

Gibson assembly uses a mixture of a 5′-exonuclease, a ligase, and DNA polymerase for one-pot isothermal assembly of overlapping DNA fragments [155]. It has been used to create a synthetic chromosome measuring several hundred kilobases, though commercially available master mixes for routine laboratory use promise high fidelity assembly of up to approximately six fragments. Gibson assembly has been used to assemble the plasmid expressing the three-component HO-BMC shell, and facilitated the switching in and out of engineered shell components to investigate cargo loading efficiency [54]. Experimentally, Gibson assembly is the most straightforward out of the three DNA assembly methodologies mentioned, requiring just incubation of PCR products with the master mix for 15 min to 1 h. Its two disadvantages are: (i) errors sometimes arise during homologous repair of overlapping regions and (ii) fragments shorter than 100 bp (such as those encoding protein linker or tag sequences) cannot be efficiently assembled [156].

### 4.2. Strategies for Installing Enzymes on Scaffolds

Methods for installing enzymes on protein and nucleic acid-based scaffolds can be broadly classified into two groups: synthetic chemistry-based and biochemistry-based. Synthetic chemistry tools make use of labile moieties on enzymes and scaffolds (e.g., terminal amines, carboxylates, sulfhydryl) to covalently link macromolecules together. The crosslinking is robust but generally not applicable for whole-cell applications due to non-specific reactions with cellular biomolecules (Table 2). Biochemistry-based tools, which include the use of ncAA/non-canonical nucleotides (ncNT), affinity tags, and protein ligases, are applicable for both in vivo and in vitro use. On the other hand, biochemical-based linking techniques may be less straightforward to carry out, or may not yield enzyme-scaffold linkages that are immune to dissociation. Ultimately, the choice of installation methodology depends on the specific enzyme and scaffold of interest.

Some widely utilized enzymes, such as GOX and HRP, are generally tolerant to chemical crosslinkers, which tend to react non-regioselectively [76,157]. It is plausible that many other enzymes suffer from major or complete loss of activity from chemical crosslinking due to such unspecific modifications. These cases are likely to be underreported in the literature. Many bio-macromolecular conjugates, discussed herein or otherwise, employ biologically benign coupling methods that afford high regioselectivity, thereby ameliorating issues in protein unfolding or inactivation of critical catalytic sites. Incorporation of ncAA or non-canonical nucleotides (ncNT) into the enzyme and scaffold allows bioorthogonal crosslinking reactions to occur intracellularly [144]. A widely used coupling reaction used is the Huisgen cycloaddition between the azido and alkynyl functional groups, termed the “click” reaction due to its robustness [144,147]. However, genetic incorporation of ncAA requires engineering/evolution of a tRNA synthetase that specifically loads a chosen ncAA onto a suppressor tRNA, while incorporation of ncNT into an oligomer similarly requires tailoring of DNA polymerases [144,158,159]. The complexity in genetically incorporating ncAA and ncNT limits their use to only the most tractable hosts, such as *E. coli* and *S. cerevisiae*.

Non-covalent affinity tags are the most widely used method for localizing foreign enzyme(s) to the scaffold. These include encapsulation or targeting peptide (EP/TP) sequences that, when installed on the cargo molecule, allow it to interact specifically with a region on the scaffold. The molecular mechanism of encapsulation/targeting varies, from docking into hydrophobic pockets observed in the *T. maritama* encapsulin [52,69], to electrostatic interactions in the *A.* *fulgidus* ferritin cage and engineered AaLS shell [78,122]. Affinity tags can be derived from proteins cognate to the compartment/scaffold, such as dockerin domains in cellulosomes, or obtained through rational design and/or evolution, such as GFP(+36) or aptamers [98,123,148]. The diversity, ease of installation, and general non-intrusive of affinity tags on the scaffold have offset their key limitation, which is detachable coupling to the scaffold. It should be noted that some affinity tags, even short ones, may have unforeseen impact on the cargo. For example, the PDU EP (18 residues long) may cause enzymes to strongly aggregate into inclusion bodies owing to hydrophobic motifs on its sequence [89].

Protein ligation techniques, in which isopeptide bonds are formed by peptide ligases, offer both high regioselectivity (due to sequence specificity of the ligases) and affinity (due to the covalent isopeptide bonds formed). The caveat is that peptide ligase domains that catalyze isopeptide bond formation between a tag and itself (e.g., SpyTag/SpyCatcher, SnoopTag/SnoopCatcher) tend to be relatively bulky for a protein fusion partner—SpyCatcher and SnoopCatcher have masses of 9 and 15 kDa, respectively. This may affect folding and supramolecular assembly of the scaffold if installed near highly structured or crowded regions. Nevertheless, structure-guided grafting of the SpyCatcher and SnoopCatcher domains into flexible regions of the scaffold protomer, such as the termini or internal loop regions, have permitted their integration into the scaffold [54,67,96,160]. External peptide ligases, such as sortase and butelase, are not integrated into the scaffold but tend to have limitations on the specific terminus at which ligation can take place [161,162]. This restricts possible spatial orientations that the enzyme can adopt in relation to the scaffold.

## 5. Conclusions and Outlook

Enzymology is an established field that is largely concerned with explaining the structure–function relationships of enzymes with regard to activity, and has more recently been expanding towards understanding the behavior of enzymes in complex and dynamic systems [163,164]. The studies discussed in this review demonstrate that the notion of controlling enzyme microenvironment via genetically manipulable scaffolds is percolating in enzymology, with direct implications for their applications in biotechnology and biomedicine. These advances have been facilitated by the cross-pollination of tools and methodologies between synthetic biology and chemical biology. The “plug-and-play” paradigm in synthetic biology has accelerated the building of multi-component enzyme-scaffold systems and downstream optimization of the components’ stoichiometries [150]. Chemical biology has provided a range of tools for regiospecific coupling of normally unrelated enzymes and scaffolds.

**Figure 14:**
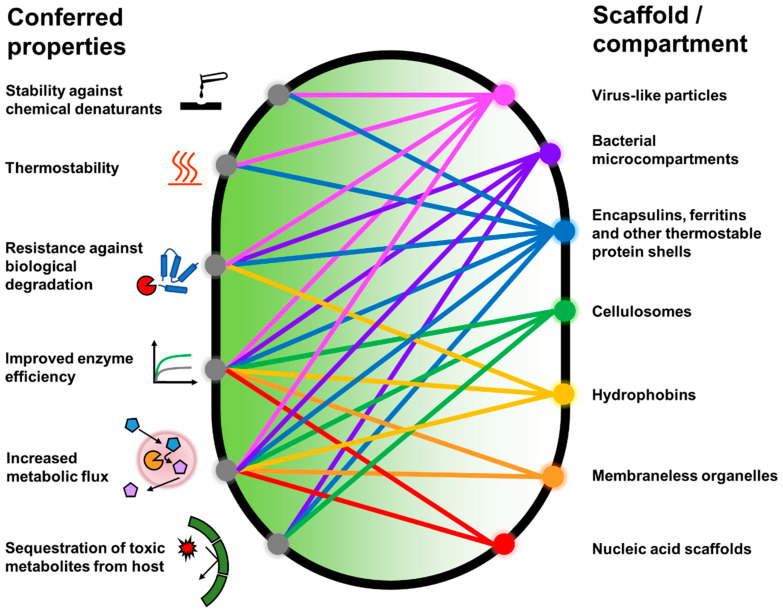
Summary of various properties in enzymes that have been conferred by the scaffolds discussed in this review.

The various protein- and nucleic acid-based scaffolds discussed herein and the desirable properties they have conferred on enzymes are summarized in Figure 14. With continuous discovery and structural elucidation of nanoscale protein shells and scaffolds, empowered by developments in genomics and structural biology, more protein-based templates can be added to the complement the current ensemble [30,68,165]. Concurrently, the burgeoning field of nucleic acid-based nanotechnology and decreasing cost of nucleic acid synthesis and modification is projected to yield more architectures capable of controlling, with greater molecular precision, the spatial orientation and activity of enzymes [131]. Two major challenges are commonly encountered by researchers when seeking to adopt protein or nucleic acid scaffolds for enhancing enzyme utility. First, despite the many molecular tools mentioned beforehand that assist in building and conjugating enzyme-scaffold systems, achieving a successful system probably still requires multiple rounds of optimization. While some scaffolds, such as MOs and homomeric encapsulin shells, can be produced by simple overexpression of the constituent modules, others, such as the multi-component BMCs and cellulosomes, require stoichiometric fine-tuning of its components [36,41,51,99]. Furthermore, the effects of protein tags and other modifications used for enzyme-scaffold coupling have to be assessed on a case-by-case basis. Synthetic biology-based methodologies that streamline combinatorial DNA assembly of individual components can alleviate part of this difficulty. Second, due to the multivariate nature of enzyme-scaffold systems, subtle biophysical properties may be overlooked in explaining the effects a specific scaffold exerts over specific enzyme(s). Consequently, desired properties reported for an enzyme-scaffold system may not necessarily be applicable should a different set of enzymes be used. As an illustration, there has been concerns that the improved activities observed in the GOX and HRP enzyme cascade widely used for prototyping DNA scaffolds, generally credited to proximity effects, may actually be caused by the acidic microenvironment in DNA scaffolds that is favored by these enzymes [132]. A wider range of enzyme cascades with more diverse reaction chemistries can be tested on existing scaffolds to provide a more substantial body of knowledge on the generalizability of altered properties in enzyme-scaffold systems.

As global demand for enzymes continues to grow in various markets, including but not limited to food, pharmaceutics, and biofuels, there is a strong motivation to engineer enzymes to fulfill emerging industrial needs [166]. To achieve economic viability, enzymes should be robust towards environments canonically uncongenial to biomacromolecules and be adaptable to industrial trends. Despite being an emerging research focus, enzyme scaffolding based on genetically encodable biomolecules has shown potential in overcoming the all-too-common bottleneck of translating research ideas to commercially viable biotechnological products.

## Figures and Tables

**Table 1:** Properties of some protein compartments and scaffolds that have been modified to host enzymes.

Protein Compartment/Scaffold	Origin	Cognate Function	Diameter/Size (nm)	Composition
Cowpea chlorotic mottle virus shell	Cowpea chlorotic mottle virus	Packaging of viral nucleic acids	28	Homomeric
Tobacco mosaic virus shell	Tobacco mosaic virus shell	Packaging of viral nucleic acids	18 (exterior) 4 (interior)	Homomeric
P22 bacteriophage head capsid	P22 bacteriophage	Packaging of viral nucleic acids	60	Homomeric
Bacterial microcompartment—carboxysome	Cyanobacteria, chemoautotrophic bacteria	Inorganic carbon fixation	~80–200	Heteromeric
Bacterial microcompartment—metabolosome	Various enteric and soil bacteria species	Catabolism of short chain carbon sources	~100–300	Heteromeric
Cellulosome	Various anaerobic bacteria species	Lignocellulose degradation	Highly variable	Heteromeric
Encapsulin	Various bacterial and archaeal species	Iron and redox metabolism	~25–30	Homomeric
Lumazine synthase	Various bacterial and fungal species	Riboflavin biosynthesis	~15–35	Homomeric
Ferritin	Bacteria, archaea, eukarya	Iron storage	~10	Homomeric
Small heat shock proteins	Bacteria, archaea, eukarya	Aids in protein refolding	~10	Homomeric
Vault ribonucleoprotein	Eukarya	Nuclear pore assembly, but exact function unclear	35 × 65	Homomeric
Hydrophobins	Filamentous fungi	Surface attachment, spore dispersion	-	Homomeric
Membraneless organelles	Eukarya	RNA processing, stress response	Highly variable	Homomeric/heteromeric

**Table 2:** Examples of widely adopted methodologies for installing enzymes onto scaffolds.

Method	Examples	Advantages	Limitations
Chemical crosslinking	*N*-hydroxysuccinimide (NHS), sulfhydryl, carbodiimide	Wide variety of reagents commercially available Covalent conjugation	Modifications are often non-regiospecific and may inactivate enzymes Incompatible with in vivo applications
Non-canonical amino acids (ncAA)/nucleotides (ncNT)	Amino acids and nucleotides containing azido, alkynyl, and other crosslinking moieties	Regiospecific and bioorthogonal Conjugation is often covalent	Inefficient and challenging incorporation of ncAA/ncNT into target molecules in vivo ncAA and ncNT are expensive or have to be synthesized in-house
Affinity tags	Encapsulation/targeting peptides, supercharged proteins (e.g., GFP(+36)), avidin-biotin, aptamers	Regiospecific Tags are often short sequences that do not severely impact protein folding/function	Dissociable linkage Tags sometimes do significantly affect protein property
Covalent protein ligation	SpyCatcher/SpyTag, SnoopCatcher/SnoopTag, peptide ligases	Covalent conjugation Regiospecific	Some protein ligation domains (e.g., SpyCatcher, SnoopCatcher) are relatively bulky and may affect protein folding/function

## Abbreviations

The following abbreviations are used in this manuscript:

AAR	Acyl-ACP reductase
ADH	Alcohol dehydrogenase
ADO	Aldehyde deformylating oxygenase
AuNP	Gold nanoparticle
BIA	Benzylisoquinoline alkaloid
BMC	Bacterial microcompartment
CBM	Carbohydrate-binding module
CPMV	Cowpea mosaic virus
CYP450	Cytochrome P450
EP	Encapsulation peptide
EPSPS	5-enolpyruvylshikimate-3-phosphate
G6pDH	Glucose-6-phosphate dehydrogenase
GCK	Gluconokinase
GFP	Green fluorescent protein
GH	Glycoside hydrolase
GOX	Glucose oxidase
GRAS	Generally regarded as safe
GSH	Glutathione
GST	Glutathione S-transferase
HRP	Horseradish peroxidase
IMAC	Immobilized metal affinity chromatography
INT	MVP interaction domain
LDH	Lactate dehydrogenase
LLPS	Liquid-liquid phase separation
MDH	Malate dehydrogenase
MnP	Manganese peroxidase
MO	Membraneless organelle
MOFS	Membraneless organelle formation sequence
MoClo	Modular cloning
MOFS	Membraneless organelle formation sequence
MVP	Major vault protein
ncAA	Non-canonical amino acid
ncNT	Non-canonical nucleotide
NHS	N-hydroxysuccinimide
NTA	Nitrilotriacetate
ORF	Open reading frame
PDC	Pyruvate decarboxylase
Pyl	Pyrrolysine
RBS	Ribosomal binding site
RE	Restriction enzyme
ROS	Reactive oxygen species
SDS	Sodium dodecylsulfate
sHSP	Small heat shock protein
TEM	Transmission electron microscopy
TMV	Tobacco mosaic virus
TP	Targeting peptide
VLP	Virus-like particle
YFP	Yellow fluorescent protein
